# Integrated Optofluidic Chip for Oscillatory Microrheology

**DOI:** 10.1038/s41598-020-62628-1

**Published:** 2020-04-02

**Authors:** Valerio Vitali, Giovanni Nava, Giuliano Zanchetta, Francesca Bragheri, Andrea Crespi, Roberto Osellame, Tommaso Bellini, Ilaria Cristiani, Paolo Minzioni

**Affiliations:** 1 University of Pavia, Dept. of Electrical, Computer and Biomedical Engineering, Pavia, 27100 Italy; 2 University of Milano, Dept. of Medical Biotechnology and Translational Medicine, Milano, 20129 Italy; 3 Istituto di Fotonica e Nanotecnologie, Consiglio Nazionale delle Ricerche (IFN-CNR), Milano, 20133 Italy; 4 Dipartimento di Fisica, Politecnico di Milano, Milano, 20133 Italy

**Keywords:** Characterization and analytical techniques, Optical manipulation and tweezers, Structure of solids and liquids

## Abstract

We propose and demonstrate an on-chip optofluidic device allowing active oscillatory microrheological measurements with sub-*μL* sample volume, low cost and high flexibility. Thanks to the use of this optofluidic microrheometer it is possible to measure the viscoelastic properties of complex fluids in the frequency range 0.01–10 Hz at different temperatures. The system is based on the optical forces exerted on a microbead by two counterpropagating infrared laser beams. The core elements of the optical part, integrated waveguides and an optical modulator, are fabricated by fs-laser writing on a glass substrate. The system performance is validated by measuring viscoelastic solutions of aqueous worm-like micelles composed by Cetylpyridinium Chloride (CPyCl) and Sodium Salicylate (NaSal).

## Introduction

Rheometry is of fundamental importance in many different fields, ranging from industrial products development to fundamental research. Assessing the different responses of a material to a mechanical stress (elasticity, viscosity, yield stress...) is of the utmost importance not only to fine tune the material response to external conditions, but also to gain a significant insight about its structure, properties and microscale structural organization^1^.

During the last decades, many high-precision rheological instruments were developed, allowing to measure the time-resolved mechanical response of a material by either imposing a controlled-strain or a controlled-stress condition. Nevertheless, an increasing attention is being paid to materials requiring to fully analyze very small sample quantities (for reasons such as high production cost, low availability of precious samples, etc...), thus pushing for the realization of new techniques able to analyze sub-*μL* samples with high reliability. This kind of analysis, generally identified as “microrheology”, is specifically attracting a large attention in the fields of bio-based and bio-inspired materials investigations^2–5^.

Microrheology exploits micrometer-sized (or nanometer-sized) beads, which get dispersed in the material under test, thus acting as tracers. The rheological properties of the material can be evaluated by monitoring the movement of tracers^6–8^. When the Brownian motion of the tracers, without the presence of any external force, is used to determine the rheological properties of the material, the technique is commonly labeled as “passive microrheology” and this approach is nowadays commonly used to perform mechanical measurements in a wide frequency range (0.1 Hz–10 kHz) on liquids and soft materials. The detection of tracers Brownian motion can be based on direct particle tracking^9,10^, on Fourier-space image analysis^11^ or on light scattering techniques^12^. The limits of passive microrheology strongly depend on the smallest tracer-displacement that can be reliably measured with a given setup, thus implying an upper limit to the maximum elastic modulus (typically a few tens of Pa) of the material under test. Furthermore, it is intrinsically limited to the investigation of linear mechanical response.

A more powerful approach (labelled “active microrheology”) is that of applying a controlled external force to actively move the tracers. Active techniques commonly rely on optical forces^13,14^ or magnetic forces^15^ to impose a known (and generally time-variant) force on the tracers that consequently stress the surrounding material. Optical forces are commonly exerted by using microscope-based optical tweezers to trap and move the tracers^16–20^. Such an approach commonly allows applying forces in a limited range going from ~0.1 pN to some tens of pN^7^, which does not allow exploring many of the situations of interest for microrheology.

Recently, a dual beam trap geometry for active microrheology was proposed^5,21^, exploiting two counterpropagating laser beams. This configuration allows obtaining stable optical trapping in a larger set of conditions when compared to microscope-based optical tweezers. In particular, the refractive index of the trapped particle can be higher than that generally allowed in conventional optical tweezers, even if some limitations (setting the maximum bead refractive index at about 5) still exist^22^. As a consequence, this configuration has the advantage of requiring a lower beam intensity while being able to apply larger optical forces with respect to conventional optical tweezers.

Such a system can be generally obtained by either assembly of discrete components^23,24^ or by realization of an integrated micro-opto-fluidic system^21,25^. In particular, using an integrated system turns out to be beneficial in terms of different aspects. The monolithic integration of the microfluidic chip and the optical waveguides ensures a good calibration and measurement repeatability since no alignment procedure is required. Additionally, the reduced size of the integrated system allows moving it easily from one lab to another in order to implement a new setup. Lastly, an integrated device ensures all the advantages of a microrheological approach such as a reduced consumption of the sample under test (even <0.1 *μL*).

In this paper we present a fully integrated microrheometer, which thanks to a custom integrated thermo-optical modulator allows a direct measurement of the material viscoelastic properties as a function of frequency in the range [0.06–60] *rad*/*s*. This system can be exploited to carry out stress-controlled active microrheology measurements in a lab-on-chip environment by accessing a wide range of optical forces, frequencies and temperatures. This allows measuring rheological properties within a significant range of viscosity and elasticity values (from dilute aqueous polymer solutions and soft gels to stiffer materials up to hundreds of Pa). The proposed device represents a significant advance with respect to a similar system we already reported^5,21^, which allowed measuring only fluids viscosity. These features make the proposed system particularly well suited for investigating the linear and non linear properties of complex media and open the way to the possibility of realizing easy to use and low cost systems for microrheometry measurements. Moreover, this device could be successfully employed to study the rheological properties and the micro-structure of heterogeneous materials.

The on-chip microrheometer is realized on a glass substrate and it embeds a square-section micro-channel with facing waveguides, lying perpendicularly to the microchannel. We show that by accurately modulating the light emitted by the waveguides by means of the custom optical modulator integrated in the setup, we are able to trap and manipulate a particle, which is subjected to a sinusoidal oscillation with a completely controllable force pattern, known in magnitude and time. The viscoelastic properties of the medium surrounding the oscillating tracer can be inferred by tracking the particle position in time by means of a camera. An interesting feature of the proposed microrheometer is that beads are trapped in the center of the channel cross-section, thus being far away from the bottom of the channel: this fact allows neglecting the effects due to the proximity of the channel walls^26^. The device performance is validated using well-studied viscoelastic samples (surfactant micellar solutions).

In the following we first give a description of the optofluidic chip and of the integrated optical modulator and we then discuss the experimental setup and procedure. Finally, the system-validation results on viscoelastic media are presented and discussed.

## Integrated system description

Optical forces provide an efficient and contact-less tool to trap and manipulate micro-objects suspended in a fluid. As already mentioned, the most common active microrheological approach to determine the viscoelastic properties of a complex fluid is based on single beam optical tweezers^7^, which rely on optical gradient forces. Conversely, we exploit the scattering forces in an integrated counterpropagating configuration to trap and subsequently sinusoidally oscillate a microbead in a microfluidic environment. The optical power emitted by the two waveguides is modulated by an electrically-driven thermo-optical modulator and the viscoelastic properties of the medium surrounding the oscillating tracer can be determined by tracking the microbead position as a function of time. The fundamental building blocks allowing to realize such a device are described in the following section.

### Working principle

The integrated microrheometer is composed by a cascade of two glass chips connected by optical fibers. The first chip consists in a Mach-Zehnder interferometer that allows modulating the optical power. The second chip contains an optofluidic system where two counterpropagating optical waveguides emit light across a microfluidic channel, thus creating the optical trap region.

**Figure 1:**
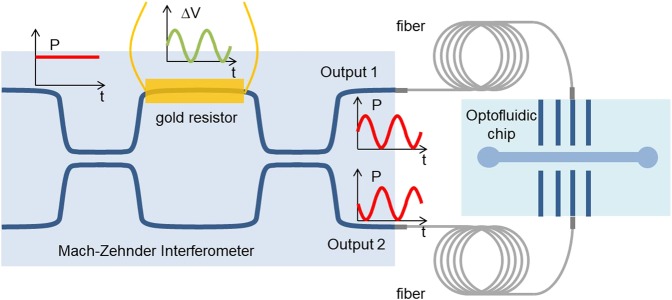
Schematic drawing of the integrated system composed by two cascaded glass chips. In the first chip, the integrated Mach-Zehnder interferometer converts a sinusoidal electrical signal in a sinusoidally oscillating optical power. The output of the modulator is fiber-pigtailed to the second chip where the oscillating optical power allows periodically moving the trapping position in the microchannel.

The interferometer is made of two cascaded 50:50 directional couplers; a gold resistor positioned near one of the two arms between the two couplers acts as an electrically-driven thermal phase-shifter (see Fig. 1). Each coupler can be considered as a beam splitter that equally divides the power in the two arms, while introducing also a *π*/2 phase term in the light that couples to the cross arm. Therefore, if the interferometer is perfectly balanced (meaning that no phase difference is produced while light propagates in the two arms), destructive interference will occur at output 1 (see Fig. 1) since the beam propagating without crossing any coupler will have phase difference of *π* compared to the light beam that crossed both couplers. On the other hand, at output 2 the two contributions will be in phase, giving constructive interference.

If a phase-shift, ranging from 0 to *π*, is accumulated along one arm, it is possible to tune the amount of optical power output by each port of the interferometer thus allowing to control the power ratio between the two output ports. A relative phase shift between the two arms can be introduced by exploiting the thermo-optic effect (i.e. a change of the refractive index induced by a temperature variation) in glass. To use such an effect, a resistive heater is realized next to one of the interferometer arms^27^.

Optical fibers connect the interferometer output waveguides to the waveguides of the optofluidic chip, thus by electrically controlling the interferometer it is possible to control the trapping position inside the microfluidic channel. As a result, by properly applying an oscillating voltage (at angular frequency *ω*) to the interferometer resistor, it is possible to produce an oscillation of the trapped bead inside the fluid at the same frequency. It is interesting to notice that, with respect to optical tweezers-based systems, such a configuration allows using beads with a higher refractive index without losing the trap stability, and exploits the so called scattering force, instead of the gradient force, and thus also allows applying much larger forces. Additionally, thanks to the possibility to trap beads with different radii (in the range 2–40 *μm*) it is also possible to investigate size effects related to the micro-structure of heterogeneous materials.

### Fabrication and characterization

Femtosecond laser micromachining is employed both to inscribe the waveguides in the two glass chips and to pattern the gold resistive heater on the modulator chip surface. As reported in^27^, the fabrication of the interferometer is a three-step process consisting in: (i) waveguides fabrication by femtosecond laser writing, (ii) deposition of a gold layer on the surface of the sample and (iii) patterning by femtosecond laser of the surface metallic layer to define the resistor shape.

The Mach-Zehnder interferometer is fabricated in an alumino-borosilicate glass substrate (Corning EAGLE-XG) by using a Yb:KYW cavity-dumped mode-locked laser oscillator that produces 300 fs pulses at 1030 nm wavelength and 1 MHz repetition rate. The waveguides are fabricated by focusing 240 nJ pulses with a 50x objective (NA = 0.6) at a depth of 30 *μ*m below the glass surface and by translating the sample at 20 mm/s (Aerotech FiberGlide translation stages). The fabricated waveguides are single mode at 1070 nm, with mode diameter of about 8 *μ*m. A complete characterization of the Mach-Zehnder optical modulator was initially carried out before its implementation in the rheological system; the device shows insertion losses, defined as the total losses introduced by the device in the setup, of about 4 dB. In order to connect the modulator to the optofluidic chip for the rheological measurements, the output waveguides are pigtailed to a fiber array. After this fabrication step, the insertion losses were estimated to be around 6.2 dB. The increase of the insertion losses is due to a mismatch between the core-to-core distance in the fiber array and the waveguide-to-waveguide distance in the chip, which makes coupling losses higher with respect to those achievable using a single-fiber pigtail. This is due to an error in the polishing step and can be therefore easily improved in future fabrications.

**Figure 2:**
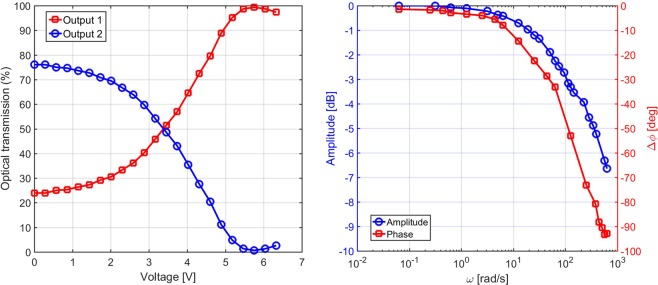
Left: optical transmission of the two arms of the Mach-Zehnder modulator as a function of the applied voltage on the resistor. The optical transmission at *V* = 0 does not reach 100% on output 2 because of couplers non-idealities. Right: amplitude- and phase-frequency response of the Mach-Zehnder modulator.

The optical transmission at the two output arms of the modulator was then measured as a function of the bias voltage applied to the resistor, which is about 125 Ω, and the results can be seen in Fig. 2 (left). This step is important in order to identify the DC-bias voltage allowing to equally split the incoming optical beam between the two output ports. The frequency response of the modulator was finally estimated by adding to the bias voltage a small (*V*_*pp*_ < 1 V) sinusoidal signal oscillating at different frequencies (up to 100 Hz). It is interesting to notice that, differently from the standard electro-optical modulators based on *χ*^(2)^ materials (such as Lithium Niobate)^28,29^, in this case the modulator transfer function is not sinusoidal with respect to the applied voltage *V*. This is due to the fact that the dissipated power, which induces the phase shift by the thermo-optic effect, depends on *V*^2^ instead of *V*. Nevertheless, in our case, when a small sinusoidal signal is added to the DC-bias, selected to obtain a 50:50 ratio between the two output ports, the transfer function is sufficiently linear to produce a sinusoidal variation of the power at the two outputs. The optical power output from the modulator was sent to a photodiode, connected to an oscilloscope in order to retrieve a voltage signal proportional to the output power. The amplitude-frequency and phase-frequency response of the modulator are shown in Fig. 2 (right). The amplitudes are normalized to the value measured at the lowest angular frequency while Δ*ϕ* is calculated as the phase difference between the voltage signal applied to the resistor and the voltage signal recorded by the photodiode. Starting from the obtained data, the −3 dB cut-off frequency was estimated to be around 17 Hz. The frequency response of the Mach-Zehnder modulator can be improved by fabricating the resistor nearer to the guiding region in order to better exploit the thermal effect that modifies the refractive index of the waveguide^30^.

The micro-opto-fluidic chip is fabricated by direct inscription of the optical waveguides in a commercial microfluidic chip (Translume Inc., Ann Arbor, MI, USA). The chip, which is also realized by fs-laser micromachining and chemical etching in a fused silica substrate, comprises a straight microfluidic channel with a square section of 150 *μ*m × 150 *μ*m and a pair of facing waveguides inscribed at half of the channel height (75 *μ*m from the floor) on the two sides of the channel. The waveguides were written by focusing 150 nJ fs-laser pulses through a 50x, 0.6 NA microscope objective and by moving the chip at a scan speed of 1 mm/s. Details about the optofluidic chip fabrication and waveguides inscription can be found elsewhere^31,32^.

## Experimental procedure

### Experimental setup

**Figure 3:**
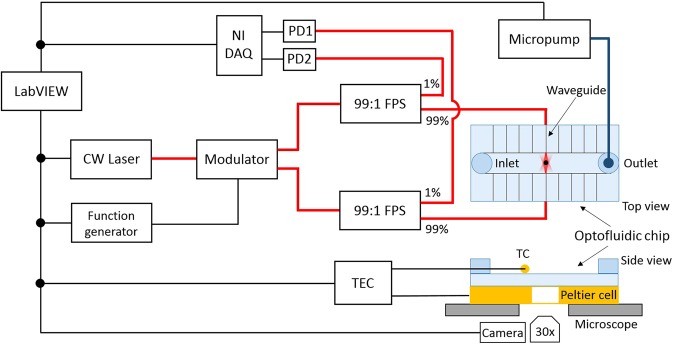
Schematic diagram of the experimental setup. The black lines represent the electrical cables connections while the thick red lines represent the optical fibers connections. TEC: temperature controller circuit; TC: thermocouple; FPS: fiber power splitter; PD: photodiode.

A schematic of the experimental setup is shown in Fig. 3. All the instruments are controlled by a custom made LabVIEW program. For what concerns the optical part, a CW Yb-doped fiber laser (YLD-10-1064, IPG Photonics, *P*_*max*_ = 10 W at 1070 nm) was employed as optical source and connected to the Mach-Zehnder optical modulator. The modulator is driven by a voltage-function generator and its two outputs are connected to a 99/1 splitter each (TN1064R1A1A Thorlabs, FPS - fiber power splitter in Fig. 3).

Fibers carrying 99% of the optical power are connected to the waveguides in the optofluidic chip, allowing to manipulate the microbeads inside the sample under test, while the 1% of the radiation is input to two photodiodes, connected to a National Instruments data acquisition system (NI DAQ in Fig. 3), allowing real-time monitoring and recording of the power levels in the two sides of the setup.

The optofluidic chip is mounted on a phase-contrast inverted microscope and the bead motion is recorded by a CMOS camera (DCC1545M Thorlabs, maximum 250 frames per second) connected to the microscope. The setup is equipped with a temperature control system, consisting of a Peltier cell placed just below the chip and a thermocouple (TC) positioned on the top of the chip, which generates the feedback signal for the Peltier cell. The pressure inside the microfluidic channel can be controlled by a LabVIEW-controlled micropump, whose tubing is connected to one of the two Luer connectors glued on the top surface of the optofluidic chip.

### Measurement protocol and data acquisition

The sample to be measured is prepared by dispersing a small amount of polystyrene microbeads (Sigma Aldrich 72986, diameter = 10 *μm*) into the fluid under test and is then injected into the microfluidic chip reservoir by a standard micro-pipette. The measurement protocol consists of the following steps. Once the sample is injected, a single microbead is trapped by the optical power emitted by the two facing waveguides and the applied pressure is adjusted by means of the micropump in order to have no flow inside the microchannel. The bias voltage of the modulator is then set so that the bead trapping position is exactly in the center of the microchannel. The temperature can be set with the temperature control system to the desired value, waiting several minutes (typically more than 10) to thermally stabilize the system.

The bead is then oscillated sinusoidally by superimposing a small sinusoidal voltage signal to the bias voltage applied to the modulator resistor. The rheological properties of the fluid under test are investigated in the range [0.06–60] *rad*/*s*. For each frequency measurement, the bead position and the voltage signals from the two photodiodes (PD1 and PD2 in Fig. 3) are acquired. The bead oscillation is monitored by a CMOS camera, which allows acquiring the particle position as a function of time thanks to a LabVIEW tracking software. The voltage signals from the two photodiodes are instead acquired by the NI DAQ.

### Fundamental equations and data processing

As discussed in the previous section, the microbead position, *x*(*t*), and the voltage signals given by the two photodiodes are recorded while oscillating the microbead at a single frequency, and the procedure is repeated several times while modifying the function generator frequency, so as to span the desired frequency-range. All the signals are acquired by a custom made LabVIEW program, which controls the whole setup. Particular attention has been paid to the synchronization of the data acquisition so as to have a proper temporal alignment of the different signals. This was accomplished by precisely measuring the time delay occurring between the acquisition of the photodiodes signals and the bead-position signal, and then introducing a proper compensation in the analysis phase. The strain *ε*(*t*) was defined starting from the microbead position as *ε*(*t*) = (*x*(*t*) − *x*_0_)/(2*R*), with *R* being the radius of the microbead and *x*_0_ the trapping position corresponding to the oscillation center. The stress acting on the microbead, *σ*(*t*), depends on the net force resulting from the difference of the forces exerted by the two counterpropagating beams on the microbead. As the voltage signals acquired by the photodiodes are proportional to the optical power carried by the two counterpropagating beams, the difference between the photodiodes signals, *V*_*ph*_(*t*), allows calculating the stress acting on the microbead, which is given by *σ*(*t*) = *β* ⋅ *V*_*ph*_(*t*), where *β* is a calibration constant. It is worth noticing that the voltage difference signal *V*_*ph*_(*t*) is a sinusoidal signal as it is given by the difference of two sinusoidal signals with the same offset value.

In order to find *β* we calibrated the system with Newtonian fluids of known viscosity *η*, as it will be discussed in the “Calibration procedure” section. As a sinusoidal modulation is applied, the optical stress acting on the microbead is of the form:

1 $$\sigma (t)=\beta \cdot {V}_{ph}(t)=\beta \cdot {V}_{ph,0}\cdot sin(\omega \cdot t)={\sigma }_{0}\cdot sin(\omega \cdot t)$$

where *V*_*ph*,0_ is the amplitude of the voltage difference signal, *ω* is the angular frequency at which the measurement is carried out and *σ*_0_ = *β* ⋅ *V*_*ph*,0_ is the stress amplitude. Assuming that measurements are performed in the linear viscoelastic regime, the resulting strain, as measured by microbead displacement, can be written as:

2 $${\varepsilon }(t)=\frac{x(t)-{x}_{0}}{2R}=\frac{A\cdot sin(\omega \cdot t-\delta )}{2R}={\varepsilon }_{0}\cdot sin(\omega \cdot t-\delta )$$

where *A* is the amplitude of the microbead oscillation, *δ* is the so-called loss angle and *ε*_0_ = *A*/(2*R*) is the strain amplitude.

The temporal evolutions of *V*_*ph*_(*t*) and *x*(*t*) are recorded over 20 periods, or more, for each single-frequency included in the investigated range. Starting from the resulting signals, it is possible to extract the values of *ε*_0_, *V*_*ph*,0_ and *δ* corresponding to the different frequencies (*ω*). Moreover, once the calibration constant *β* is determined, *σ*_0_ can be immediately calculated.

This allows calculating the “complex compliance” J^*^, which naturally describes the viscoelastic properties of the system under test when stress-controlled deformations are applied:

3 $${J}^{\ast }={J}^{{\prime} }-i\cdot {J}^{{\prime\prime} }=\frac{{\varepsilon }_{0}}{{\sigma }_{0}}(cos(\delta )-i\cdot sin(\delta ))$$

where the term ${J}^{{\prime} }$ is called “storage compliance” and accounts for the elastic contribution while the term *J*″ is the “loss compliance” and takes into account the viscous part. A good description for several viscoelastic fluids with a single relaxation time and low frequency viscous behaviour is provided by the Maxwell model, which can be represented by a purely elastic spring and a purely viscous damper connected in series^1^. By considering the constitutive equations describing the Maxwell model^1^, the complex compliance can be written as:

4 $${J}^{\ast }={J}^{{\prime} }-i\cdot {J}^{{\prime\prime} }=\frac{1}{E}-i\cdot \frac{1}{\omega \eta }$$

where E is the elastic modulus and *η* is the viscosity. Starting from these two parameters, it is possible to calculate the relaxation time, setting the crossover between the two behaviours, as *η*/*E*.

Finally, the rheological properties of the system under study can be expressed in an analogous way by calculating the “complex modulus” ${G}^{\ast }$ :

5 $${G}^{\ast }=\frac{1}{{J}^{\ast }}={G}^{{\prime} }+i\cdot {G}^{{\prime\prime} }$$

where the term ${G}^{{\prime} }$ is the “storage modulus” and accounts for the elastic contribution while the term ${G}^{{\prime\prime} }$ is the “loss modulus” and accounts for the viscous contribution.

### Laser-induced heating

As viscoelastic properties can be strongly dependent on temperature, we evaluated the laser-induced heating effect inside the microchannel, following an approach analogous to those already reported in the literature^33^. The sample temperature during oscillatory measurements is estimated to be equal to ${T}^{{\rm{{\prime} }}}=T+\Delta T$, where *T* is the temperature measured by the thermocouple and Δ*T* is the temperature increase due to the laser heating, which depends on the optical power in the microfluidic channel and on the sample under test. In order to determine Δ*T* for aqueous solutions, the temperature-dependent fluorescence of Rhodamine B was used^33,34^. An aqueous solution of 0.1 mM Rhodamine B was prepared and injected in the microchannel. Fluorescence imaging of the dye was performed by using a mercury arc lamp (Nikon C-SHG1) and an appropriate filter cube (TxRed, excitation: 540–580 nm; emission: 600–660 nm). A calibration curve was derived to determine the temperature dependence of Rhodamine B fluorescence intensity in our system. This was realized by carrying out measurements of fluorescence emission intensity at different temperatures (in the range 18–45 °C) by actuating the Peltier cell, while the laser radiation was switched off during this procedure. Laser-induced heating was then estimated by measuring the fluorescence intensity variation in the center of the microchannel. Considering an optical power of 0.5 W in the microchannel, matching the optical power used in the oscillatory experiments reported in the following, we observed a laser-induced heating *ΔT* = (5 ± 1) °C. For this reason, a fixed laser-induced offset of 5 °C, with respect to the temperature measured by the thermocouple, was considered to evaluate the sample temperature (${T}^{{\rm{{\prime} }}}$). It is worth noticing that the estimated temperature increase is in line with the value of (13 ± 2) °C/W previously reported by Ebert *et al*.^33^ in a similar dual beam laser trap geometry.

### Calibration procedure

The calibration constant *β* can be determined by measuring fluids of known rheological properties such as water or water-glycerol mixtures. In case of water, the complex compliance reduces to its imaginary part *J*″, which can be written as:

6 $${J}^{{\prime\prime} }=\frac{{\varepsilon }_{0}}{{\sigma }_{0}}\cdot sin(\delta )=\frac{{\varepsilon }_{0}}{\beta \cdot {V}_{ph,0}}$$

where *sin*(*δ*) = 1 due to the purely viscous nature of water. From Eq. (4) we have that *J*″ = 1/(*ωη*_*water*_) and by combining it with Eq. (6) it is possible to calculate the calibration constant *β* as:

7 $$\beta =\frac{{\varepsilon }_{0}}{{V}_{ph,0}}\cdot \omega {\eta }_{water}$$

Measurements performed in water at several angular frequencies *ω* and at different optical power levels yielded the same calibration constant *β*. More specifically, the uncertainty of the *β* value, calculated as the ratio of *β* standard deviation with respect to its average value, was measured to be equal to 1.5%. It is worth noticing that both *ε*_0_ and *V*_*ph*,0_ are directly proportional to the optical power, thus making *β* to be power independent. The calibration measurements performed in water also confirmed the aforementioned assumption *sin*(*δ*) = 1, with a measured loss angle *δ* = 90° ± 1° (see Supplementary Fig. S2). The whole calibration procedure was carried out in water using a low optical power level (<100 mW) so as to limit the laser-induced heating effect, which could yield an over estimation of the optical power in the microfluidic system. The calibration constant depends on geometrical data of the light beam, the radius *R* and the refractive index *n*_*P*_ of the particle and the refractive index *n*_*X*_ of the sample under test. While the first three parameters are fixed for all the experiments that use the same device and particles, *n*_*X*_ can obviously vary as materials with different refractive indices can be tested. As *n*_*X*_ significantly affects three parameters (the single-photon momentum, the reflection coefficient at the bead surface and the beam divergence), numerical simulations based on paraxial ray-optics (PRO) approach^13,35^ were used to calculate the appropriate correction factor. As demonstrated by Ferrara *et al*.^35^, numerical simulations based on PRO allow a high-precision calculation of the optical forces exerted by a non-focused Gaussian laser beam on a dieletric microbead and thus the error introduced by numerical simulations in the valuation of the appropriate force is negligible with respect to other sources of error, such as the uncertainty in the microbead position (see Supplementary Information). When studying the rheological properties at different length-scales is important, the proposed method offers as a natural possibility that of changing the bead radius. In this case, as the calibration constant *β* is experimentally determined for a given microbead radius, numerical simulations must be used to calculate the appropriate correction factors according to the radius of used microbeads.

### Sample under test

Some surfactant molecules in solution can self-assemble into elongated, cylindrical micelles, called “worm-like micelles” (WLMs)^36^, which show distinct rheological features and are widely used in a variety of consumer products. WLMs show many analogies to covalently bonded polymers, but they also continuously break and reform and are therefore called “living polymers”. Above a critical concentration of surfactant or counterion/surfactant ratio, the micelles start to overlap and then form an entangled network, which thus displays the typical rheological features of a Maxwell viscoelastic fluid. Its mechanical response is mainly elastic at high frequency, while at low frequency the system can rearrange and mainly behaves like a Newtonian fluid. The relaxation time separating the two regimes results from the combination of two mechanisms for stress relaxation: reptation, the constrained diffusion of a micelle within the tube of the neighbouring micelles, and reversible breakage^37^. To validate the proposed system, we thus tested the performance of our setup for oscillating microrheology by using a viscoelastic solution of surfactant worm-like micelles as a benchmark material. To this aim, we prepared solutions using Cetylpyridinium Chloride (CPyCl) as a surfactant and Sodium Salicylate (NaSal) as a counterion in milliQ water at twice the final concentration and then mixed them at equal volumes to obtain 100 mM CPyCl/50 mM NaSal and 100 mM CPyCl/55 mM NaSal. All the chemicals were bought from Sigma Aldrich. Solutions were gently stirred for more than 24h and then stored at ambient temperature. The samples remained stable over a few weeks.

## Results

**Figure 4:**
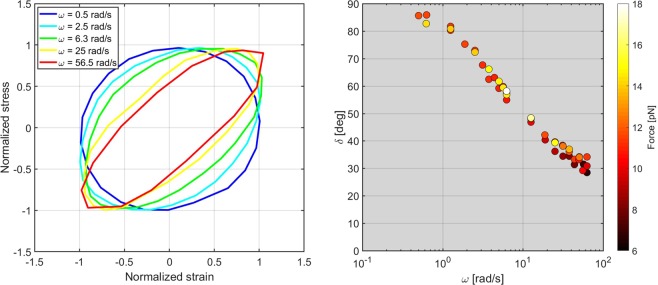
Results from the measurements of an aqueous worm-like micellar solution of 100 mM CPyCl/50 mM NaSal at an estimated sample temperature ${T}^{{\rm{{\prime} }}}$= 29 °C. Left: Lissajous figures for different angular frequencies. The stress during a complete oscillation is plotted as a function of the strain, for five different frequency values. In order to reduce the error, the reported stress and strain signals were obtained by calculating the average signals out of a minimum of 20 periods; we also normalized the signals to their maximum amplitude, so as to highlight the change of ellipticity of the Lissajous figures. Right: Loss angle *δ* as a function of angular frequency and of optical force.

Measurements were performed keeping the system at controlled temperature thanks to the presence of the Peltier controller. CPyCl/NaSal displays a well characterized viscoelastic behaviour^36,38^, which can be used to validate the proposed measurement system. Oscillatory experiments were initially carried out on a 100 mM CPyCl/50 mM NaSal sample keeping the thermocouple temperature equal to *T* = 24 °C. Considering the average laser-induced heating *ΔT* = 5 °C, the sample temperature was estimated to be ${T}^{{\rm{{\prime} }}}$= 29 °C. As expected, the system displays different regimes depending on the applied frequency, as can be appreciated in the stress-strain (Lissajous) plots in Fig. 4 (left).

**Figure 5:**
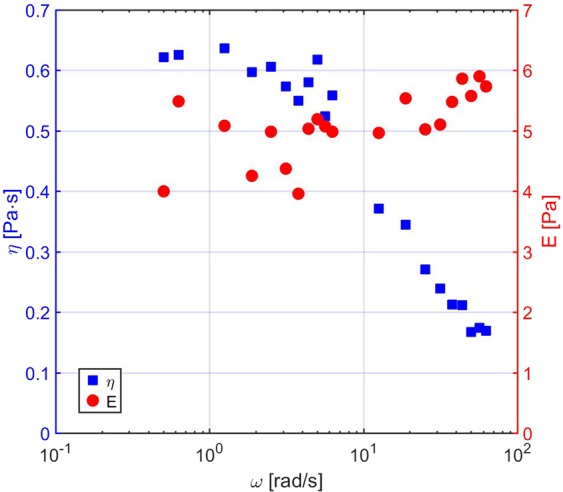
Results from the measurements of an aqueous worm-like micellar solution of 100 mM CPyCl/50 mM NaSal at an estimated sample temperature ${T}^{{\rm{{\prime} }}}$= 29 °C. Viscosity *η* (blue squares) and modulus *E* (red circles) as a function of angular frequency.

At low frequency, the micellar solution responds as a purely viscous fluid (a circle in the stress-strain plane), while at high frequency (>20 rad/s) the response has a strong elastic, in-phase component, shown by the fact that the Lissajous plots become tilted, elongated ellipses. As described by the previously reported equations, starting from the stress vs. strain data (Lissajous plots), we can extract the phase *δ*, the elastic modulus *E* and the viscosity *η* as a function of *ω*. The loss angle *δ* (see Eq. (2)) as a function of *ω*, shown in Fig. 4 (right), quantifies the transition between the two regimes, varying from almost 90° at low *ω* (viscosity-dominated regime) to less than 30° at high *ω* (elasticity-dominated regime). By considering the Maxwell model for describing the viscoelastic behaviour of this system, it is possible to derive the *ω*-dependence of the viscosity *η* and of the elastic modulus *E*, which are shown in Fig. 5. As expected, for increasing frequency, elasticity increases and viscosity decreases.

The bead size is much larger than the mesh-size of the micellar network (few nm in our case) and the limit on strain linearity simply depends on the relative motion of the bead^1^. As in our experiments the oscillation amplitude of the bead was always observed to be within a few % of its diameter, we expect that our measured values lie within the linear viscoelastic response of the WLM network. Nevertheless, in order to experimentally verify the system linearity, we performed the experiments at different frequencies for various optical forces. Different optical forces were imposed by changing the amplitude of the small sinusoidal signal applied to the modulator resistor while keeping the optical power in the microchannel equal to 0.5 W. In Fig. 4 (right) we show that both the phase *δ* and its dependence on frequency are not affected by the applied forces, thus confirming that all the results belong to the linear-response regime of the material under test. In addition, by performing a Fourier analysis of the position signal, the amplitudes of the higher harmonics were found to be comparable to the noise level and always lower than the 2% of the amplitude associated to the fundamental oscillation frequency, thus further confirming the linearity of the material response.

## Discussion

**Figure 6:**
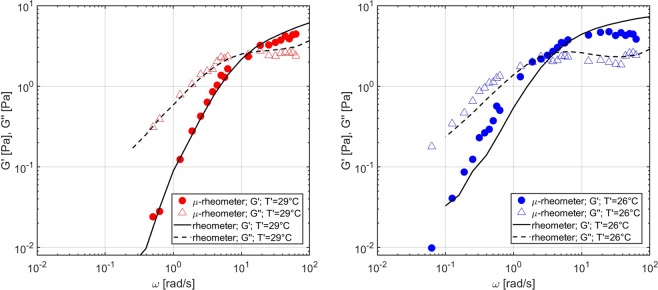
Results from the measurements of aqueous worm-like micellar solutions. Left: Complex modulus of a 100 mM CPyCl/50 mM NaSal micellar solution at an estimated sample temperature ${T}^{{\rm{{\prime} }}}$ = 29 °C. Right: Complex modulus of a 100 mM CPyCl/50 mM NaSal micellar solution at ${T}^{{\rm{{\prime} }}}$ = 26 °C. The microrheological measurements were compared with the results obtained using a conventional rheometer and a good agreement was found.

When converting our data in storage and loss moduli, ${G}^{{\prime} }$ and ${G}^{{\prime\prime} }$ (see Fig. 6 (left)), we find that at low frequency *G*″ ∝ *ω* is dominant over ${G}^{{\prime} }\propto {\omega }^{2}$, as expected for a Maxwell fluid. Above the crossover frequency, the elastic component becomes dominant over the viscous one and ${G}^{{\prime} }$ approaches a plateau value. The measurement uncertainty, calculated as the ratio of ${G}^{{\prime} }$ and ${G}^{{\prime\prime} }$ standard deviation with respect to their average values, was found to be around 12% on average, considering the different explored frequencies, and always lower than 20%. For this reason, no error bar is reported in Fig. 6, where a logarithmic scale is used. The same samples have been measured with a standard rheometer (Anton Paar MCR 302) for comparison. The estimated scaling behaviour, the absolute values of the relaxation time and of storage and loss moduli closely match values obtained from macroscopic rheology measurements performed at the same temperature of 29 °C.

**Figure 7:**
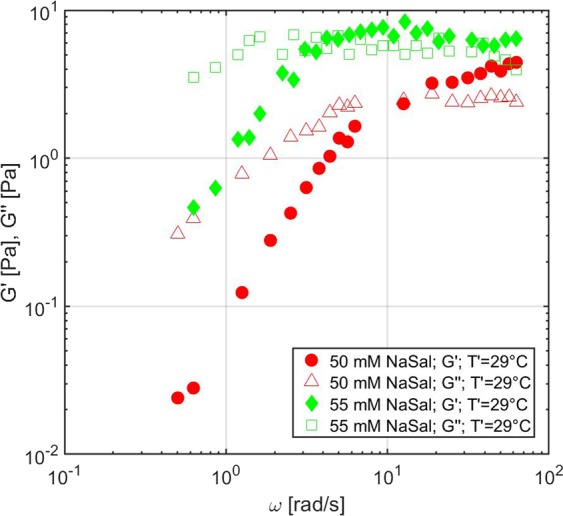
Results from the measurements of aqueous worm-like micellar solutions. Complex modulus of two different micellar solutions (100 mM CPyCl/50 mM NaSal and 100 mM CPyCl/55 mM NaSal) measured at the same sample temperature ${T}^{{\rm{{\prime} }}}$ = 29 °C.

Changes in surfactant concentration, type and concentration of counterions, or temperature can significantly affect the length and flexibility of micelles, which therefore affect the elasticity and the relaxation time of the network^36^. The results from the measurements performed on the same micellar sample at a different temperature are shown in Fig. 6 (right). Specifically, the Peltier module in the microrheological setup was actuated to reach a stable thermocouple temperature of 21 °C. Considering the laser-induced heating effect, the sample temperature was estimated to be ${T}^{{\rm{{\prime} }}}$= 26 °C. As it can be observed, decreasing the temperature shifts the relaxation time to a higher value, because it increases the average time for micellar breakage; instead, it has almost no effect on the elastic plateau, which mainly depends on the mesh size of the network, unaffected by the increased average length of the micelles. The microrheological measurements were compared with the results obtained from a conventional rheometer set at the same temperature ${T}^{{\rm{{\prime} }}}$ and a good agreement was found even in this situation. Further measurements were then carried out by increasing the concentration of counterions on a 100 mM CPyCl/55 mM NaSal sample and were compared with those performed on the 100 mM CPyCl/50 mM NaSal sample at the same sample temperature ${T}^{{\prime} }$= 29 °C. Increasing the concentration of counterions partially screens the electrostatic repulsion between micelles, and thus increases the energetic cost of scission. This makes the micelles longer and more flexible, thus increasing the relaxation time and decreasing the mesh size, and therefore increasing the value of the elastic plateau, as it can be observed in Fig. 7. Even in this case, our measured trends are in agreement with previously reported measurements on the same micellar system^38^.

## Conclusions

An on-chip optical microrheometer based on a dual beam laser trap is reported. The device allows performing active oscillatory microrheological measurements in the frequency range from 0.01 Hz to 10 Hz with a very low sample consumption (<1 *μL*) and with temperature setting capability. The device consists of a Mach-Zehnder optical modulator connected to an optofluidic chip, where the power emitted by two facing femtosecond-laser inscribed waveguides allows manipulating the position of a trapped microbead in the microfluidic channel. Trapped microbeads can be oscillated sinusoidally thanks to the actuation of the optical modulator, thus making possible to retrieve the viscoelastic properties of the fluid under test. System calibration was achieved by measuring simple Newtonian fluids. The microrheometer was validated by measuring the viscoelastic parameters of aqueous worm-like micellar solutions of CPyCl/NaSal, at different concentrations and at different temperatures, and comparing the obtained results with those derived using a conventional rheometer and with data already reported in the scientific literature. The strongest limitations to the maximum frequency (10 Hz for our measurements) are due to the low camera frame rate, the use of a relatively slow LabVIEW cycle and to the −3 dB cut-off frequency of the Mach-Zehnder modulator, thus clearly indicating the possibility to improve system performance by optimizing those elements. In addition to the low sample consumption, the high integration level of the microrheometer provides multiple benefits. The monolithic integration of the chip ensures an excellent calibration and measurement repeatability as no alignment of optical components is required, differently from microscope-based optical tweezers. The setup implementation is straightforward and the chip portability gives the possibility to easily move the microrheological setup from one lab to another. Finally, the precise control and the wide range of achievable optical forces ensure a flexible use of the device, making it possible to carry out stress-controlled active measurements, in linear or non linear regime, on a wide range of materials, from aqueous polymer solutions and soft gels to stiffer materials, thus opening the way to the development of a miniaturized, automatized and fully-integrated device.

## Supplementary information

Supplementary Information.
