# Supplementary material for: Integrated Optofluidic Chip for Oscillatory Microrheology
Source: Sci Rep. 2020 Apr 2;10:5831. doi: 10.1038/s41598-020-62628-1 (PMC7118116; doi:10.1038/s41598-020-62628-1)
Supplement: Supplementary file 1 — Supplementary Information. [file 41598_2020_62628_MOESM1_ESM.pdf]

Integrated Optofluidic Chip for Oscillatory Microrheology

Valerio Vitali[1], Giovanni Nava[2], Giuliano Zanchetta[2], Francesca Bragheri[3], Andrea Crespi[3, 4], Roberto Osellame[3, 4], Tommaso Bellini[2], Ilaria Cristiani[1], and Paolo Minzioni[1,*]

[1]University of Pavia, Dept. of Electrical, Computer and Biomedical Engineering, Pavia, 27100 Italy
[2]University of Milano, Dept. of Medical Biotechnology and Translational Medicine, Milano, 20129, Italy
[3]Istituto di Fotonica e Nanotecnologie, Consiglio Nazionale delle Ricerche (IFN-CNR), Milano, 20133, Italy
[4]Dipartimento di Fisica, Politecnico di Milano, Milano, 20133, Italy
[*]paolo.minzioni@unipv.it

Supplementary information

Mach-Zehnder modulator: working principle

The Mach-Zehnder modulator is a classical building block, used in different optical devices and a fundamental component in optical communication systems. A laser source sends a beam, of optical power P_0, into an optical fiber which is then aligned in front of an optical waveguide (blue line in the drawing) created in the substrate material.

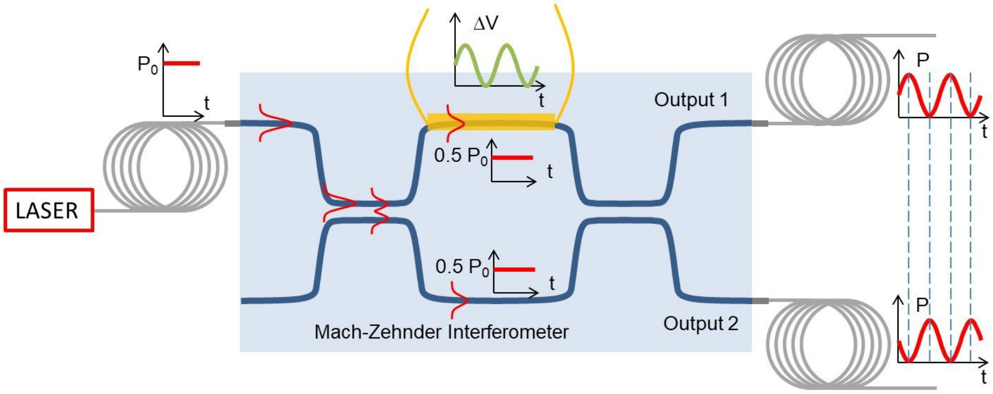

Figure S1. Conceptual drawing of the Mach-Zehnder interferometer. For sake of clarity, dimensions are not to scale. The light emitted by the laser source is coupled to the integrated waveguides (blue lines) by a fiber (shown in grey). A voltage applied to the gold resistor allows changing the distribution of the input power between the two output waveguides.

The light beam, spatially having a Gaussian intensity distribution (typical of the waveguide fundamental mode), is guided till the area where two waveguides are brought in close proximity, thus creating a so called "coupling region". In this area, the tail of the input beam has a non negligible overlap with the second waveguide, and hence the input beam power gets gradually coupled from the first to the second waveguide. The coupling-ratio (i.e. the ratio between the optical power of the beams propagating in the two waveguides) produced in this region depends on the distance between the two waveguides and on the length of the coupling region. In our study, the Mach-Zehnder coupling regions were designed so as to evenly split the power among the two waveguides. In absence of any phase shifter (the gold electrode in our case) the two propagating beams would accumulate exactly the same phase shift along the waveguides before reaching the second coupling region, and in that case they would produce destructive interference at output 1 (yielding the absence of optical power) and constructive interference at output 2, thus resulting in a 100% transfer of the optical power from the input waveguide to output 2. The reason why, in case of a "balanced interferometer" (i.e. a perfectly symmetrical device), the light would be coupled to output 2 instead of output 1 is that when light gets coupled from one waveguide to the other it also acquires a $\pi/2$ phase term. The presence of a gold resistor can then be used to locally change the temperature on just one side ("arm") of the structure, by applying a temporally varying electrical current. This produces a change of the local refractive index and thus a variation of the phase acquired by the optical beam propagating in the top waveguide. The phase-change then causes, in the second coupling region, a modification of

the interference condition, thus allowing to control the amount of power transferred to the two output waveguides.

Extraction of rheological quantities and error propagation analysis

As discussed in the *Fundamental equations and data processing* section, when small stress-controlled deformations are applied, the rheological properties of the material under test are described by the "complex compliance" J^*:

$$J^* = J' - i \cdot J'' = \frac{\varepsilon_0}{\sigma_0}(cos(\delta) - i \cdot sin(\delta)) \tag{S1}$$

where ε_0 is the strain amplitude, σ_0 is the stress amplitude and δ is the loss angle. Once the complex compliance is determined, it is possible to calculate all the rheological quantities of interest such as the viscosity, elasticity and complex modulus, as discussed in the main text. By recalling that $\varepsilon_0 = A/(2R)$ and $\sigma_0 = \beta \cdot V_{ph,0}$, it is possible to rewrite eq. S1 as:

$$J^* = J' - i \cdot J'' = \frac{A}{2R\beta \cdot V_{ph,0}}(cos(\delta) - i \cdot sin(\delta)) \tag{S2}$$

where A is the amplitude of the bead oscillation, R is the bead radius, β is the calibration constant and $V_{ph,0}$ is the amplitude of the voltage difference signal. In order to give an estimation of the error in the measurement of the complex compliance, it is possible to add in quadrature the variation coefficients affecting the parameters appearing in eq. S2. Concerning the bead position, it was detected with an accuracy of 10 nm by real-time image processing using a LabView tracking algorithm. The variation coefficient of bead oscillation amplitude A equal to 3% and 9% was calculated at the lowest and highest oscillation frequency, respectively. A 2% uncertainty is considered to affect the radius of polystyrene microbeads used in the experiments (Sigma Aldrich 72986, diameter = 10 μm), as by product data sheet. By analyzing the calibration measurements performed in water, it was possible to assess the uncertainty of the β value to be 1.5%, and the uncertainty in the calculation of the functions $cos(\delta)$ and $sin(\delta)$ in below 1%. Finally, the variation coefficient affecting the measurement of $V_{ph,0}$ was measured to be equal to 0.5% and 2% for low and high oscillation frequency, respectively. Summing in quadrature all the contributions, the variation coefficient in the measurement of the real part J' and imaginary part J'' of the complex compliance J^*, and hence of the rheological properties of the material under test, was estimated to be comprised between 4% (at the lowest frequency) and 9.6% (at the highest frequency).

Microrheometer calibration: water measurements

The results of the microrheometer calibration measurements performed in water, whose procedure is described in the *Calibration procedure* section, are reported in Fig. S2. In particular, Fig. S2 (left) shows the measured loss angle δ, while Fig. S2 (right) reports the measured calibration factor β. As it can be observed, the calibration measurements carried out in water confirmed the assumption $sin(\delta) = 1$ and the independence of the calibration factor β on the angular frequency ω.

Calibration verification: glycerol measurements

Using the value of β obtained from the water calibration measurements, a sample of pure glycerol was then measured using polystyrene microbeads (Sigma Aldrich 72986, diameter = 10 μm) at a temperature of 23 °C and at different angular frequencies in order to verify the calibration procedure. Having glycerol a different refractive index (1.463) with respect to water, the force was corrected as discussed in the *Calibration procedure* section by means of numerical simulations based on paraxial ray-optics (PRO) approach[1]. The results can be seen in Fig. S3. The measured viscosity was found to be $\eta = 1.066 \pm 0.012\ Pa \cdot s$ in good agreement with the expected one for pure glycerol $\eta_{literature} = 1.078\ Pa \cdot s$ at a temperature of 23 °C[2]. As expected, being glycerol a purely viscous material, the loss angle δ is constant around 90°(see Fig. S3 (left)) and the viscosity is independent from the angular frequency (see Fig. S3 (right)).

References

1. Ferrara, L. *et al.* Experimental study of the optical forces exerted by a gaussian beam within the rayleigh range. *J. Opt.* **13**, 075712 (2011).

2. Cheng, N.-S. Formula for the viscosity of a glycerol- water mixture. *Ind. & engineering chemistry research* **47**, 3285–3288 (2008).

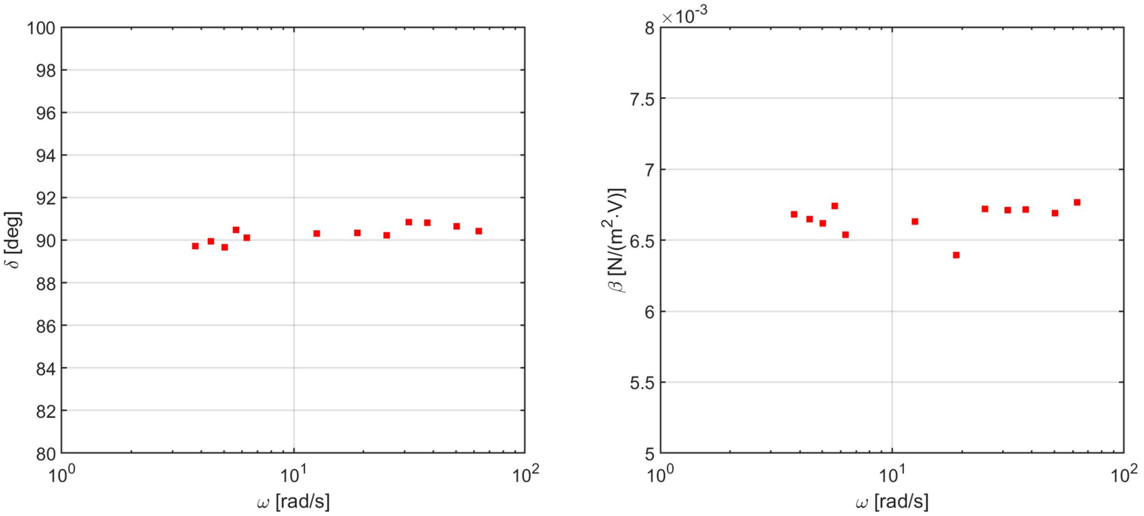

Figure S2. Results from the calibration measurements performed in water. Left: Loss angle δ as a function of different angular frequencies ω. The calibration measurements performed in water confirmed the assumption $sin(\delta) = 1$. Right: Calibration factor β calculated for different angular frequencies ω.

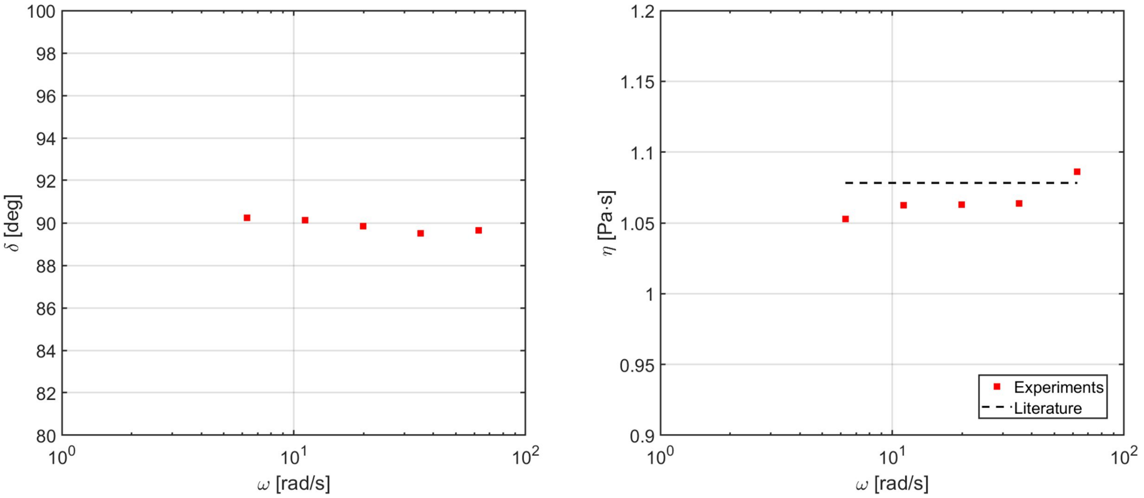

Figure S3. Results from the measurements of pure glycerol. Left: Loss angle δ as a function of different angular frequencies ω. Right: Resulting viscosity η (red squares) of pure glycerol measured at 23 °C as a function of angular frequency ω and expected viscosity from scientific literature (black dashed line)
